# Supplementary material for: Cerebellar repetitive transcranial magnetic stimulation has no effect on contraction-induced facilitation of corticospinal excitability
Source: PLoS One. 2024 Nov 1;19(11):e0310173. doi: 10.1371/journal.pone.0310173 (PMC11530076; doi:10.1371/journal.pone.0310173)
Supplement: Attachment — Submitted filename: Response to Reviewers.docx [file pone.0310173.s001.docx]

**Response to Editors’ and reviewers’ comments**

We greatly appreciate the helpful suggestions concerning our manuscript titled “No effect of cerebellar repetitive transcranial magnetic stimulation on contraction-induced facilitation of corticospinal excitability”. We have addressed all the concerns of the editor and reviewer, and have carefully revised our manuscript accordingly. The changes are marked using track changes. Below are our point-by-point responses to the reviewers’ comments. Please let us know if further revisions are needed; we would be glad to incorporate them.

**Summary of Revisions**

**Reviewer 1**

**(Comment #1)**

While the introduction effectively outlines the general role of the cerebellum, the rationale for investigating CIF of corticospinal excitability within the context of this cerebellar study remains unclear. CIF represents a unique condition wherein subjects are required to sustain muscle contraction, which may not reflect a natural physiological state. Given this, it is essential for the authors to clarify why CIF was chosen as a focal point in this cerebellar study and elucidate its significance. Specifically, understanding the motivation behind investigating CIF and its relevance to cerebellar function and clinical implications would strengthen the rationale for the study design. Additionally, providing a thorough explanation of the physiological background underlying CIF and its implications for cerebellar modulation of corticospinal excitability would enhance the interpretation of the study's findings.

**(Reply to Comment #1)**

Thank you very much for your valuable feedback. We understand the need to clarify the rationale for investigating CIF in the context of our current study, and have majorly revised the introduction section.

**(Comment #2)**

Some subjects exhibited CBI ratios exceeding 1 during pre-rTMS contraction (Fig.2b, red circles). CBI is defined as MEP inhibition (<1) in response to conditioned stimuli relative to MEP amplitude during test stimuli. It is imperative for the authors to elucidate the reasons behind this occurrence and explain why these subjects were not excluded, especially in light of the criteria used to exclude two subjects due to resting CBI. If this occurrence is validated, I would inquire about the physiological implications of such a finding in relation to the study outcomes.

**(Reply to Comment #2)**

Thank you for your comment. The inhibition of MEP by conditioning stimulation of the cerebellum at rest was certainly confirmed before the experiment, however, CBI at contraction condition was not confirmed before the experiment. Figure 2b shows there were no participants in the absence of CBI at rest in the pre-stimulation condition. This finding consists of inclusion criteria for participants with experiments about CBI at rest. On the other hand, the red circles indicate the contraction conditions, and this condition’s CBI was not checked before the experiment.

**(Comment #3)**

Of greater concern is the statistical disparity between the one-sample test and linear mixed model analyses. The former suggested that rTMS reduced CBI during the resting state, while no significant effects on CIF, rMT, cSP, and CBI during muscle contraction were observed (The null hypothesis for CBI is equal to 1, indicating no inhibition; however, it is important to clarify whether this is tested using one-sided or two-sided). Conversely, the latter statistical approach did not corroborate these findings, indicating no effects of time, TMS intervention, or contraction on CBI. Given these inconsistent results, the authors arbitrarily concluded a positive effect of rTMS on the cerebellum at rest, and a negative effect on motor function during muscle contraction. However, the statistical validity of these conclusions is debatable. In addition, it is imperative for the authors to carefully elaborate on the interpretation of negative findings, particularly the absence of rTMS effects on the cerebellum during muscle contraction. Several potential pitfalls, including technical issues related to data acquisition (coil parameters) and statistical power, should be thoroughly addressed.

**(Reply to Comment #3)**

Thank you for your great suggestive advice. As the reviewer said, the interpretation of the statistical results in this study is difficult and we think it also needs to be interpreted with careful considerations.

Regarding CBI, no significant suppression was observed only in the post-active rTMS condition, which could be due to the effect of rTMS. On the other hand, no significant differences were observed between the groups divided by factors in the liner mixed model analysis, suggesting that rTMS did not have an effect that caused a significant difference between the groups in terms of CBI and CIF. It is very reasonable to assume that if there is no difference between groups in the liner mixed model analysis, there should be the same suppression in the one-sample t-test as in the pre, but this was not the case in our results.

In general, when examining the effects of multiple factors using ANOVA or linear mixed models, significant differences are less likely to emerge as the number of groups compared increases. On the other hand, simple two-group comparisons have a relatively lower risk rate. The differences in the statistical methods used in the linear mixed model analysis and the one-sample t-test in this study may have led to apparently contradictory results.

Linear mixed model analysis was used to test the effect of each factor on CIF and CBI because we wanted to minimise the influence of potentially confounding subjects, but this analysis alone could not test whether a CIF or CBI had been achieved. Therefore, we used one-sample t-test under two-sided was used to test the CIF and CBI were obtained, and we conservatively set the null hypothesis for CIF and CBI are not equal to 1. As both analyses were performed conservatively, we believe that the results themselves are valid. Therefore, we believe that the implications of these results should be interpreted with caution.

We have described the lack of consistency in the results of this statistical approach as a limitation. As the reviewers pointed out, we made sure to include a discussion of statistical power in the first place. We also described the effects of stimulus parameters that may have occurred during data acquisition as a point of concern.

The effects observed for CBI at rest but not during muscle contraction were discussed on the basis of possible mechanisms in the third paragraph of the Discussion section.

**Reviewer 2**

**(Comment #1)**

As a general impression, the manuscript could benefit from a (complete) rewriting by a native speaker, a professional writer, or an AI tool (just one example among many, L.45-47 "...evidence of the effects of cerebellar degeneration and modulation of cerebellar output excitability in the motor cortex or corticospinal excitability during force control remains lacking." is intelligible but quite awkward to read. This observation unfortunately applies to almost every single phrase in the manuscript)

**(Reply to Comment #1)**

Thank you for your helpful comment. This manuscript was re-checked and re-edited by English language editing service ([www.editage.jp](http://www.editage.jp)).

**(Comment #2)**

The study is well-designed and conducted, and the conclusions drawn are sound. The observation that 1Hz rTMS over the cerebellum can reduce cerebellar inhibitory output towards M1, yet does not modulate the excitability of the contralateral M1, is particularly relevant given that this effect remains debated. However, a major question arises concerning the manuscript's suggestion to use NIBS (specifically rTMS) for modulating cerebellar output in SCD patients: what are the bases for expecting that the cerebellar circuits in SCD patients might respond to NIBS in a manner similar to those of healthy individuals? This is suggested towards the end of the discussion (L349-359).

**(Reply to Comment #2)**

Thank you for your comment. This paragraph was structured in a way that made our explanation very difficult for the reader to understand. We have substantially revised the text of this paragraph to make our explanation easier to understand, based on the reviewer's comment.

**(Comment #3)**

The authors might also want to address the following minor points:

1. An increased RMT does not necessarily imply suppression of corticospinal excitability.

**(Reply to Comment #3)**

Thank you for your comment. As the reviewer mentioned, the increase in rMT is not due to a single factor. We have added that there are several possibilities.

**(Comment #4)**

2. Please avoid repeating information from the main text in the figure legends; choose one location for each piece of information.

**(Reply to Comment #4)**

Thank you for your suggestion. We have changed the legends to avoid duplication.

**(Comment #5)**

3. Use the explicit description "1Hz rTMS" instead of the more general "low-frequency" rTMS

**(Reply to Comment #5)**

Thank you for your suggestion. We have changed from “low-frequency rTMS” to “1 Hz rTMS” in those areas where more specific wording is appropriate.

We have carefully considered your comment and have made major revisions to the manuscript. Thank you again for your valuable and constructive contributions to our research. We are looking forward to hearing from you.

Best regards
