# Supplementary material for: Cerebellar repetitive transcranial magnetic stimulation has no effect on contraction-induced facilitation of corticospinal excitability
Source: PLoS One. 2024 Nov 1;19(11):e0310173. doi: 10.1371/journal.pone.0310173 (PMC11530076; doi:10.1371/journal.pone.0310173)
Supplement: Attachment — Submitted filename: Response to Reviewers_R3_20240806.docx [file pone.0310173.s002.docx]

**Response to Editors’ and reviewers’ comments**

We greatly appreciate the helpful suggestions concerning our manuscript titled “Cerebellar repetitive transcranial magnetic stimulation has no effect on contraction-induced facilitation of corticospinal excitability”. We have addressed all the concerns of the editor and reviewer, and have carefully revised our manuscript accordingly. The changes are marked using track changes. Below are our point-by-point responses to the reviewers’ comments. Please let us know if further revisions are needed; we would be glad to incorporate them.

**Summary of Revisions**

**Reviewer 1**

**(Comment #1)**

(1) As previously mentioned, the physiological meaning of CBI exceeding 1 during contraction is unclear. While CBI exhibits inhibitory function at rest, the authors do not explain its physiological significance during contraction. Approving such occurrences in multiple subjects across different conditions (sham/active, pre/post), the statistical analysis was conducted assuming CBI is below 1 on both sides. Please explain why a one-sided t-test is valid in a setting where several subjects with CBI exceeding 1 during contraction are not excluded.

**(Reply to Comment #1)**

Thank you very much for your valuable feedback. Previous studies have also reported that CBI does not occur during muscle contraction (Kassavetis et al. 2011, and Kassavetis et al. 2016), but the mechanism is not yet clear. We have added a hypothesis to the Discussion about the possible mechanism, which is also discussed in previous studies (lines 395-403 in revised manuscript with track changes).

Since the exclusion criterion is no-observation of CBI at rest, the exclusion criterion is not met even if CBI does not occur during muscle contraction. Therefore, in the contraction condition, cases exceeding 1 cannot be excluded. Such cases exceeding 1 during contraction are expected to be included from the range of error bars presented in the graphs of previous studies (Kassavetis et al. 2011, and Kassavetis et al. 2016).

The test we used is a two-sides test for CBI: we are testing the null hypothesis that CBI is not 1. We did not used one-side test of one-sample test. It was already described in the methods (lines 215-216), but for greater clarity, it was also stated in the results that it was a two-tailed test (line 285).

**(Comment #2)**

(2) If my understanding is correct, for measuring CBI, the test stimuli were delivered at 90% of the rMT in relation to the primary motor cortex (P11, line 196). Does this provide sufficient MEP amplitude to evaluate CBI accurately? Additionally, what intensity was the conditioning stimulus delivered to stimulate the cerebellum after measuring the rMT for cerebellar TMS? Was there any change in the rMT for the cerebellum after rTMS, considering that 1-Hz rTMS over the cerebellum immediately reduced motor function (P11, line 205)?

I recommend that the authors show illustrative examples of the changes or lack thereof in CBI, CIF, and cSP.

**(Reply to Comment #2)**

Thank you very much for your valuable feedback. This is completely error of our mention. "the left primary motor cortex" should be corrected to "the right cerebellum". To induce test MEP on right FDI muscle, we stimulate the left primary motor cortex, and the stimulation intensity used in this experiment was set to elicit a test MEP size of 0.5–1 mV to measure CBI before experiment. This explanation had been described in previous section (Single-pulse TMS over the primary motor cortex (test stimulation)) (lines 171-174).

For cerebellar stimulation, the intensity was set to 90% of rMT with double cone coil on right cerebellum. The rMT for cerebellar TMS was defined as the lowest stimulation intensity producing a short-latency EMG response and cervicomedullary MEP in the right FDI muscle immediately after cerebellar TMS for five of ten consecutive stimuli (Tanaka et al. 2017). This explanation had been described in same section (Single-pulse TMS over the cerebellum (conditioning stimulation)) (lines 187-192).

The rMT for the cerebellum was not significantly changed after rTMS. This result was described in Result section ((a) rMT) (lines 237-242).

We added the typical waveform of MEP as Fig3.

We believe that your advice has made the results of this study more understandable to our readers. Thank you once again for your suggestable advice.

**(Comment #3)**

(3) Please provide an explanation of the null hypothesis regarding the one-sample t-test in cSP. The data analysis section does not describe that a one-sample t-test was conducted for cSP, but the results section showed these results. If this is valid, please provide p values in Table 5. Additionally, please add an interpretation in the discussion regarding the findings from the linear mixed model, which showed a significant fixed effect of time and interaction between time and TMS conditioning.

**(Reply to Comment #3)**

We are very sorry; we did not apply the one-sample t-test to the cSP; the table of averages etc. output mechanically by JASP was included as is. This is what is described in Fig. 2(d), so table5 has been deleted.

We did not attend to the importance of this interaction until the reviewers pointed it out to us. To further clarify the effect of this interaction, we conducted a post-hoc comparison. The results showed no effect of conditioning stimulation, only a clear effect of time. In the previous version of the manuscript, the discussion of the cSP results was only included as the limitation, but considering the results of this additional analysis, a new paragraph has been added to the discussion (lines 436-443). In adding the statistical analysis, the methods (lines 220-221) and results (lines 343-347) have also been changed. Again, thanks to the reviewers' remarks, our errors were corrected, and we were able to add additional analyses that provide further evidence. We would like to thank them once again.

**(Comment #4)**

(4) Overall, I would recommend emphasizing the statistical considerations in this paper, particularly the negative results regarding the cerebellar contribution to CIF. I especially consider the marginal results on the interaction between time and rTMS in CIF (p = 0.058) in the linear mixed-effect model. In addition, the Figure 3c implies this interaction. The one-sample t-test for CIF does not make much sense to me because the null hypothesis may include not only two sides (<1 indicating inhibition, >1 indicating facilitation) but also the change of its magnitude after rTMS. The negative finding may not necessarily indicate the lack of physiological effect. I would recommend emphasizing the methodological limitations regarding this once again.

**(Reply to Comment #1)**

Thank you very much for your valuable feedback, and we agree this idea. We majorly revised discussion section with adding one paragraph for explanation on these concerns (lines 425-435).

**(Comment #5)**

(5) In Figure. 1b, please specify the intensity of cerebellar stimulation in relation to the rMT of the cerebellum used to generate this electrical field, and provide evidence supporting its validity for stimulating/modulating the cerebellum within this electrical field.

**(Reply to Comment #5)**

Thank you for your comment. The rMT for cervicomedullary MEP in Sham and Active-rTMS group at pre- and post-rTMS were 36.3 ± 7.04 % (mean ± standard deviation), 34.9 ± 4.04 %, 36.2 ± 7.13 %, and 34.5 ± 4.09 %, respectively. The electrical field of Fig1(b) was generated by SimNIBS software in the case of using maximum intensity using D-B80 coil connected to magnetic stimulator (MagVenture). The mean of rMT was about 35% of maximum output, indicating about 1.23*0.35*0.9=0.387 V/m may be delivered to cerebellar cortex. The data used for this calculation are described in Methods (line 218), Results (lines 237-242) and further discussion in paragraph of limitation (lines 451-455).

**(Comment #6)**

Minor comments

P11, line 199: "To measure CBI, the optimal ISI at which maximal MEP inhibition was selected was 4-9 ms." The word "selected" should be replaced with "observed."

P11, line 214: "Finally, rTMS analysis was performed..." The term "analysis" should be replaced with "intervention" or a similar term.

**(Reply to Comment #1)**

Thank you for your point out our mistake of choice the terms. These terms were revised based on the reviewers’ comments.

We have carefully considered your comment and have made major revisions to the manuscript. Thank you again for your valuable and constructive contributions to our research. We are looking forward to hearing from you.

Best regards
